# A CpG Methylation Signature as a Potential Marker for Early Diagnosis of Hepatocellular Carcinoma From HBV-Related Liver Disease Using Multiplex Bisulfite Sequencing

**DOI:** 10.3389/fonc.2021.756326

**Published:** 2021-10-20

**Authors:** Kang Li, Yi Song, Ling Qin, Ang Li, Sanjie Jiang, Lei Ren, Chaoran Zang, Jianping Sun, Yan Zhao, Yonghong Zhang

**Affiliations:** ^1^ Biomedical Information Center, Beijing You’An Hospital, Capital Medical University, Beijing, China; ^2^ Experimental Center, Beijing Friendship Hospital, Capital Medical University, Beijing, China; ^3^ St. Edmunds College, Cambridge, United Kingdom; ^4^ Pharmacology Department, Air Force Medical Center, People’s Liberation Army of China (PLA), Beijing, China; ^5^ Clinical Laboratory Center, Beijing You’An Hospital, Capital Medical University, Beijing, China

**Keywords:** multiplex bisulfite sequencing, CpG methylation, early HCC, diagnostic model, enhanced bootstrap validation

## Abstract

**Background:**

Aberrant methylation of CpG sites served as an epigenetic marker for building diagnostic, prognostic, and recurrence models for hepatocellular carcinoma (HCC).

**Methods:**

Using Illumina 450K and EPIC Beadchip, we identified 34 CpG sites in peripheral blood mononuclear cell (PBMC) DNA that were differentially methylated in early HCC versus HBV-related liver diseases (HBVLD). We employed multiplex bisulfite sequencing (MBS) based on next-generation sequencing (NGS) to measure methylation of 34 CpG sites in PBMC DNA from 654 patients that were divided into a training set (*n* = 442) and a test set (*n* = 212). Using the training set, we selected and built a six-CpG-scorer (namely, cg14171514, cg07721852, cg05166871, cg18087306, cg05213896, and cg18772205), applying least absolute shrinkage and selection operator (LASSO) regression. We performed multivariable analyses of four candidate risk predictors (namely, six-CpG-scorer, age, sex, and AFP level), using 20 times imputation of missing data, non-linearly transformed, and backwards feature selection with logistic regression. The final model’s regression coefficients were calculated according to “Rubin’s Rules”. The diagnostic accuracy of the model was internally validated with a 10,000 bootstrap validation dataset and then applied to the test set for validation.

**Results:**

The area under the receiver operating characteristic curve (AUROC) of the model was 0.81 (95% CI, 0.77–0.85) and it showed good calibration and decision curve analysis. Using enhanced bootstrap validation, adjusted C-statistics and adjusted Brier score were 0.809 and 0.199, respectively. The model also showed an AUROC value of 0.84 (95% CI 0.79–0.88) of diagnosis for early HCC in the test set.

**Conclusions:**

Our model based on the six-CpG-scorer was a reliable diagnosis tool for early HCC from HBVLD. The usage of the MBS method can realize large-scale detection of CpG sites in clinical diagnosis of early HCC and benefit the majority of patients.

## Introduction

Hepatocellular carcinoma (HCC) is the most common primary liver tumor with high morbidity and mortality. It has been estimated that hepatitis B virus (HBV) infection causes 50%–80% of HCC cases worldwide (1). The progression of multi-stage hepatocarcinogenesis from chronic HBV infection (CHB) to HBV-related liver cirrhosis (LC) and finally to HBV-related HCC is complex. There is 2%–7% incidence of LC per year in CHB patients (2), and 2%–4% annual incidence of HCC in LC patients (3). Meanwhile, CHB patients can also develop HCC without cirrhosis. The new data show that annual HCC rates ranged 0.03%–1.57% among non-LC Asian CHB male patients (4). In involving 34,952 patients, the study suggested that 2.29% of CHB patients would develop HCC despite hepatitis B surface antigen (HBsAg) seroclearance (5). There are several mechanisms related with HBV-related hepatocarcinogenesis progression including viral regulatory HBV × protein interrupting liver cell proliferation and increasing HBV replication (6), integration of HBV DNA into the host cell genome provoking host cell chromosomal alterations and insertional mutagenesis of cancer genes (7), and host genomic and epigenetic aberrant variation induced by inflammation (8).

The high mortality rate of HCC is mostly due to its discovery at advanced stages. There is an urgent unmet need for biomarkers that can detect early HCC development in CHB and LC liver background. DNA methylation profiles for early stage of HCC in peripheral blood mononuclear cells (PBMC) are different from healthy controls as well as CHB. HCC has a specific DNA methylation signature in easily accessible PBMC and can serve as “noninvasive” biomarkers for detection of early HCC as well as HCC progression (9). Several studies have demonstrated CpG methylation prepared for constructing diagnostic, prognostic, and recurrence models for HCC (10–12).

However, in these models, measurement methylation of CpGs was performed by pyrosequencing or methylation specific PCR. These methods were laborious and involved fussy work (13) for detection methylation of dozens of CpGs and inevitable increase of inter-batch differences in large samples. This could cause adverse effects on the diagnostic efficacy of the model.

Therefore, in this study, we applied multiplex bisulfite sequencing (MBS), which is based on the next-generation sequencing (NGS) method to assess the methylation status of 34 CpGs in 654 samples at the same time. Multivariable methods identified a minimal set of CpGs to achieve optimal prediction. Meanwhile, we handled multiple imputation to complete missing data and bootstrapped training datasets for backward feature selection. The model incorporated both the six-CpG-scorer methylation panel and demographic and clinical characteristics risk factors for early diagnosis onset of HCC from HBV-related liver disease (HBVLD) including CHB and LC.

## Materials and Methods

### Data Processing for CpG Selection

The raw IDAT files of Illumina 450K Beadchip data including CHB patients (*n* = 10) and Barcelona clinic liver cancer staging system (BCLC) 0 (*n* = 10), BCLC-A (*n* = 10), BCLC-B (*n* = 10), and BCLC-C (*n* = 9) were obtained from Gene Expression Omnibus (accession number: GSE67170) (9), which was prepared to obtain differentially methylated CpGs between CHB and each HCC stage. Forty-four paired PBMC samples were taken from 22 patients before and again after diagnosis of HCC. The 22 patients were diagnosed as LC prior to HCC, and then 5 were diagnosed as HCC BCLC-0 and 17 were BCLC-A. Twenty-two LC and 22 early HCC PBMC samples were subjected to genome-wide DNA methylation assay by using Illumina EPIC Beadchip (data did not published). The R package ChAMP was applied for methylation raw data processing and the paired t tests were used to analyze the differentially methylated CpGs between LC and HCC BCLC-0 and -A. The differentially methylated CpGs with |delta beta|≥ 0.2 and remaining significant after Bonferroni-corrected *p* value < 0.05 were selected.

### Patient Study Population

The cross-sectional retrospective study, using the convenient sampling method, was conducted from August 2010 to July 2019 at Beijing You’An Hospital, which is an infectious diseases specialist tertiary hospital in China. A total of 654 patients were included in this study. The demographic and clinical characteristic data of patients with CHB, LC, and early HCC were collected from hospital electronic medical records. The inclusion criterion of CHB was that hepatitis B surface antigen (HBsAg) seropositive status lasted at 6 months or beyond according to the Asia-Pacific clinical practice guidelines on the management of hepatitis B: a 2015 update (14). Diagnosis of hepatitis B-related LC was based on a combination of clinical, laboratory, imaging features, and liver biopsies according to the guideline of prevention and treatment for chronic hepatitis B (2010 Version). Diagnosis of HCC was based on the radiological/histological criteria according to 2012 EASL clinical practice guidelines: Management of chronic hepatitis B virus infection, Barcelona clinic liver cancer staging system (BCLC) (15). Exclusion criteria were as follows: less than 18 years old; co-infection with human immunodeficiency virus, hepatitis C virus, or hepatitis D virus; coexistence of liver injury caused by drug intake, alcohol consumption, and autoimmune hepatitis; pregnancy; and lactation. The study was approved by the Institutional Ethics Committee of the Beijing You’An Hospital. All participants signed written informed consent on enrolment.

### DNA Bisulfite Converted and Multiplex Bisulfite Sequencing

After extraction, PBMC DNA bisulfite was converted with sodium bisulfite according to the protocol of EZ DNA Methylation-Direct Kit (ZYMO Research, Irvine, USA), whose bisulfite conversion rate was >99.5%. Bisulfite-transformed DNA was a single strand and we measured the concentration of the single-strand DNA using a Qubit 2.0 (Thermo, USA, catalog Q10212) to ensure that adequate amounts of single-template DNA were invested for library construction. A panel contained 34 “CpG-free” primer pairs designed to target all candidate CpGs. Library preparation was performed by nest-PCR. First-round PCR reaction was set up as follows: bisulfite converted DNA 5 μl; forward primer mix (10 μM) 1 μl; reverse primer mix (10 μM) 1 μl; 2×PCR Ready Mix 15 μl (total 25 μl) (KAPA HiFi HotStart ReadyMix PCR). The plate was sealed and PCR was performed in a thermal instrument (BIO-RAD, T100TM) using the following program: 1 cycle of denaturing at 98°C for 3 min, then 27 cycles of denaturing at 98°C for 20 s, annealing at 60°C for 4 min, and final elongation at 72°C for 2 min. Finally, hold at 10°C. The PCR products were checked using electrophoresis in 1% (w/v) agarose gels in TBE buffer (Tris, boric acid, EDTA) stained with ethidium bromide (EB) and visualized under UV light. Then, we used AMPure XP beads to purify the amplicon product. After that, the second-round PCR was performed. PCR reaction was set up as follows: first-round PCR amplicon product (10 ng/μl) 2 μl; universal P7 primer with barcode (10 μM) 1 μl; universal P5 primer with barcode (10 μM) 1 μl; 2×PCR Ready Mix 15 μl (total 30 μl) (Kapa HiFi Ready Mix). The plate was sealed and PCR was performed in a thermal instrument (BIO-RAD, T100TM) using the following program: 1 cycle of denaturing at 98°C for 1 min, then eight cycles of denaturing at 98°C for 20 s, annealing at 60°C for 20 s, elongation at 72°C for 30 s, and a final extension at 72°C for 2 min. Then, we used AMPure XP beads to purify the second-round PCR amplicon product. The libraries were then quantified and pooled. Paired-end sequencing of the library was performed on the HiSeq XTen sequencers (Illumina, San Diego, CA).

### Data QC and SNP Calling

MBS raw data were processed by Sangon Biotech. Briefly, raw reads were filtered according to three steps: (a) removing adaptor sequence if reads contains by cutadapt (v 1.2.1); (b) removing low quality bases from reads 3’ to 5’ (Q < 20) by PRINSEQ-lite (v 0.20.3); (c) Bismark (version v0.22.1) (www.bioinformatics.babraham.ac.uk/projects/) was used for CpGs detected with default parameters.

### CpG Selection and Assessment

**Figure 1:**
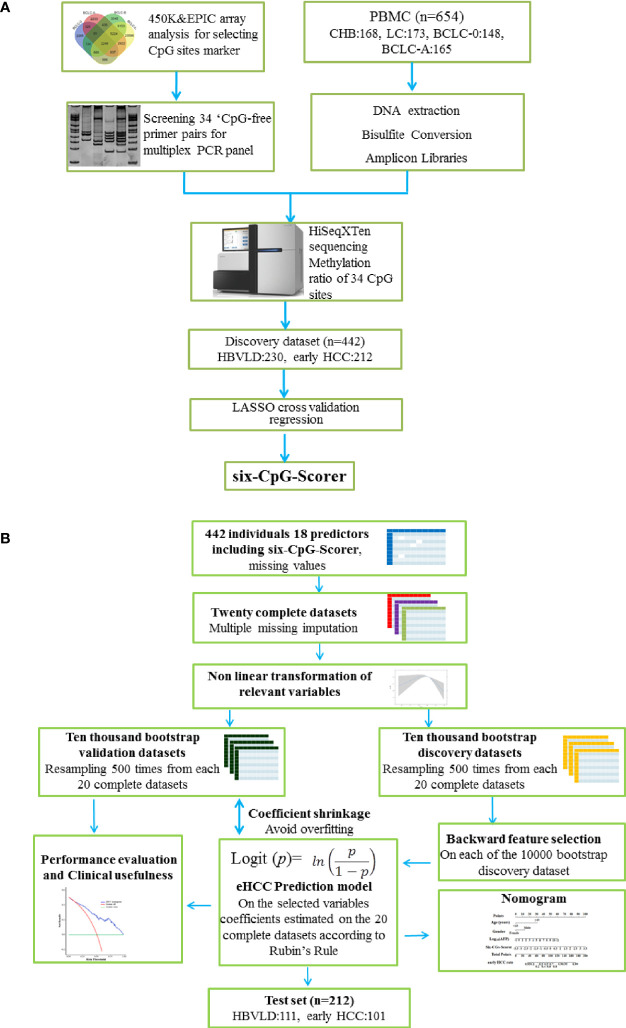
Study design and analysis protocol. **(A)** Study design and flow diagram. A total of 654 PBMC samples were prospectively collected. Thirty-four CpGs were investigated using multiplex bisulfite sequencing in 341 HBVLD (CHB: 168, LC: 173) and 313 early HCC group (BCLC-0:148, BCLC-A: 165). The least absolute shrinkage and selection operator method (LASSO) cross-validation was introduced to selected six CpGs and built a six-CpG-scorer in the training dataset (*n* = 442, 212 early HCC and 230 HBVLD). **(B)** Schematic representation of statistical analysis. The analyses were performed on 18 selected predictors measured on 442 parents involved in the training set. Twenty complete datasets were created after 20 times missing imputation, and 17 candidate variables were detected the possible nonlinear dependency of the relationship with early HCC, of which 7 variables were transformed in a non-linear fashion. Resampling 500 times were from each of the 20 complete datasets, leading to a total number of 10,000 bootstrap datasets. A backward feature selection with AIC was repeated on each of the 10,000 bootstrap datasets for selecting the most relevant risk variables for early HCC. The factors chosen at least once during the feature selection procedure constituted the final mode, whose coefficients were estimated using Rubin’s Rule from the 20 complete datasets. In internal validation, the model predictiveness and correcting overfit were assessed using 10,000 separate enhanced bootstrapped datasets. The nomogram presented the final mode. Decision curve analysis and clinical impact curves were performed to determine the final model clinical usefulness. The final mode also showed an obvious diagnosis potential in test set (*n* = 212, 101 early HCC and 111 HBVLD). HBVLD, HBV-related liver disease; HCC, hepatocellular carcinoma; BCLC, Barcelona Clinic Liver Cancer staging system.

A multi-CpG-based scorer was constructed to diagnose the early HCC patients from HBVLD in the training dataset. The least absolute shrinkage and selection operator (LASSO) with cross-validation method was applied to select the most significant CpGs from the 34 described in **Supplementary Table S1**. The process was mainly performed using the “glmnet” package (16) based on the R software (Version 4.0.3) and depicted in **Figure 1A**.

### Statistical Analysis

Firstly, the restricted cubic splines (RCS) were applied to detect the possible nonlinear dependency of the relationship between early HCC and 17 continuous variables, using three knots at the 25th, 50th, and 75th percentiles of the corresponding variable. There were potential threshold associations between early HCC and five indices [age, direct bilirubin (DBil), total protein (TP), albumin (ALB), and hemoglobin] (*p*-value for nonlinear < 0.001). We have categorized age to the following groups:<43 years and ≥43 years, DBil to <7 μmol/L and ≥7 μmol/L, TP to <65 g/L and ≥65 g/L, ALB to <42 g/L and ≥42 g/L, and hemoglobin to <140 g/L and ≥140 g/L based on the RCS curve. The other four indices [total bilirubin (TBil), γ-glutamyltranspeptidase (γ-GT), platelet count (PLT), and lymphocyte count (LYM)] were categorized to normal and abnormal groups based on the medical reference value, respectively. The other seven indices [alanine aminotransferase (ALT), aspartate aminotransferase (AST), alkaline phosphatase (ALP), white blood cell count (WBC), monocyte count (MONO), neutrophil count (NUET), and alpha-fetoprotein (AFP)] were log-transformed, respectively (**Supplementary Table S1**).

Missing values in original dataset were imputed 20 times using imputation with predictive mean matching (“mice” package) (17). For each of the 20 complete dataset, 500 bootstrapping datasets were generated, and backwards feature selection with the Akaike Information Criterion (AIC) was performed on the 10,000 datasets (“rms”) (18). Second-order interaction terms were also tested for inclusion. The selected predictors constructed the final model, whose regression coefficients were calculated using “psfmi” package according to “Rubin’s Rules” (19, 20). The enhanced bootstrap method was to evaluate the stability of the diagnosis model. Discrimination ability was assessed by the area under the receiver operating characteristic curve (AUROC) and C-statistics. Calibration was applied for assessment of agreement between predicted and observed risks across of the population. The nomogram presented the results of the modeling process. Decision curve analysis (DCA) was performed to determine the model clinical usefulness. The construction process referred to this report (21) and the model analysis conforms to the reporting standards of STARD (22) and was depicted in **Figure 1B**.

## Results

### Demographic and Characteristics of the Patients

After a strict pathological diagnosis and exclusion process, 168 patients with CHB, 173 patients with liver cirrhosis, 148 patients with HCC BCLC-0 stage, and 165 patients with HCC BCLC-A stage were collected in Beijing You’An hospital and included into this study (**Figure 1A**). A total of 654 patients were randomly assigned to the training set (*n* = 442) and test set (*n* = 212). Demographic and clinical characteristics of patients are shown in **Table 1**.

**Table 1:** Clinical characteristics of the enrolled participants in this study (*n* = 654).

	Training set (442)		Test set (212)	
	HBVLD (*n* = 230)	Early HCC (*n* = 212)	HBVLD (*n* = 111)	Early HCC (*n* = 101)
Age (years, mean ± SD)	45.37 ± 11.9	46.22 ± 11.7	45.38 ± 12.08	52.24 ± 10.36
Sex (M/F)	170/60	174/38	80/31	90/11
ALT (U/L)				
<40	108 (47.0%)	128 (60.4%)	51 (45.9%)	60 (59.4%)
≥40	121 (52.6%)	83 (39.2%)	60 (54.1%)	41 (40.6%)
AST (U/L)				
<40	125 (54.3%)	140 (66.0%)	59 (53.2%)	55 (54.5%)
≥40	104 (45.2%)	71 (30.9%)	52 (46.8%)	46 (44.5%)
Total bilirubin (μmol/L)				
<21	144 (62.6%)	147 (69.3%)	79 (71.1%)	63 (62.4%)
≥21	85 (37.0%)	64 (30.2%)	32 (18.9%)	38 (37.6%)
Direct bilirubin (μmol/L)				
<7	169 (73.5%)	175 (82.5%)	88 (79.3%)	72 (71.3%)
≥7	60 (26.1%)	36 (17.0%)	23 (20.7%)	29 (28.7%)
Total protein (g/L)				
<65	168 (73.4%)	135 (59.0%)	85 (76.6%)	65 (64.4%)
≥65	61 (26.5%)	76 (35.8%)	26 (23.4%)	36 (35.6%)
Albumin (g/L)				
<40	136 (59.1%)	105 (49.5%)	72 (64.9%)	45 (44.6%)
≥40	93 (40.4%)	106 (50.0%)	39 (35.1%)	56 (55.4%)
γ-GT (U/L)				
<45	121 (52.6%)	95 (44.8%)	52 (46.8%)	40 (39.6%)
≥45	96 (41.7%)	95 (44.8%)	52 (46.8%)	61 (60.4%)
Alkaline phosphatase (U/L)				
≤100	172 (74.8%)	142 (67.0%)	83 (74.8%)	48 (47.5%)
>100	46 (20.0%)	48 (22.6%)	22 (19.8%)	37 (36.6%)
WBC count × 10^9^/L				
<3.5	174 (75.7%)	157 (74.1%)	79 (71.2%)	74 (73.3%)
3.5–9.5	46 (20.0%)	42 (19.8%)	24 (21.6%)	14 (13.9%)
>9.5	10 (4.3%)	12 (5.7%)	8 (7.2%)	13 (12.9%)
Hemoglobin (g/L)				
<130	143 (62.2%)	138 (65.1%)	77 (69.4%)	60 (59.4%)
≥130	87 (37.8%)	73 (34.4%)	34 (30.6%)	41 (40.6%)
Platelet count × 10^9^/L				
<125	129 (56.1%)	108 (50.9%)	70 (63.1%)	58 (57.4%)
≥125	101 (43.9%)	104 (49.1%)	41 (36.9%)	43 (42.6%)
Lymphocyte count × 10^9^/L				
<1.1	172 (74.8%)	149 (70.3%)	85 (76.6%)	58 (57.4%)
≥1.1	58 (25.2%)	62 (29.2%)	26 (23.4%)	43 (42.6%)
Monocyte count × 10^9^/L				
<0.6	214 (93.1%)	182 (85.8%)	107 (96.4%)	81 (80.2%)
≥0.6	16 (6.9%)	29 (13.7%)	4 (3.6%)	20 (19.8%)
Neutrophil count × 10^9^/L				
<1.8	166 (72.2%)	167 (78.8%)	80 (72.1%)	71 (70.3%)
1.8–6.3	53 (23.0%)	17 (8.0%)	26 (23.4%)	12 (11.9%)
>6.3	11 (4.8%)	17 (8.0%)	5 (4.5%)	18 (17.8%)
Alpha-fetoprotein (ng/ml)				
<20	178 (77.4%)	110 (51.9%)	86 (77.5%)	41 (40.6%)
≥20	50 (21.7%)	96 (45.3%)	25 (22.5%)	58 (57.4%)

HBVLD, HBV-related liver disease; CHB, chronic hepatitis B; LC, HBV-related liver cirrhosis; HCC, hepatocellular carcinoma; BCLC, Barcelona clinic liver cancer staging system; γ-GT, γ-glutamyltranspeptidase; WBC, white blood cell. ALT, alanine aminotransferase; AST, aspartate aminotransferase.

### CpG Selection and Panel Signature Building for Early HCC Diagnosis

**Figure 2:**
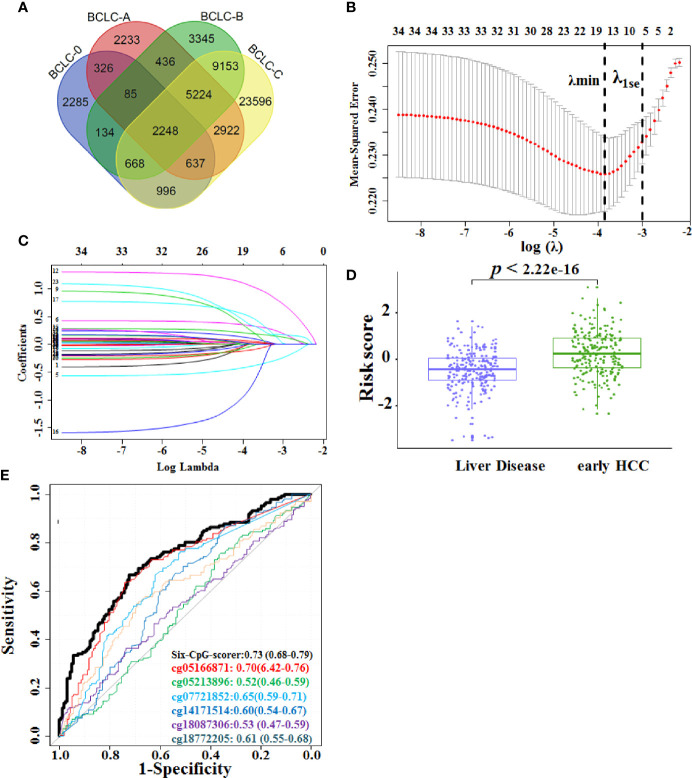
Construction of the six-CpG-scorer in training set. **(A)** Venn diagram of the overlap between differentially methylated CpGs between different HCC BCLC stages (0-C) and CHB. **(B)** Fivefold CV LASSO coefficients of 34 candidate CpGs. The first black vertical dotted line corresponds to the λmin that minimized binomial deviance during CV. The second black vertical dotted line corresponds to λ1se, used for the selection of six biomarker CpGs. **(C)** LASSO coefficient profile of the 34 early HCC-associated CpGs. A vertical line was drawn at the value chosen by fivefold cross-validation. **(D)** The six-CpG-scorer of each participant from the liver disease, early HCC group in the training dataset was calculated, and the score was significantly increased in the early HCC versus the HBV-related liver disease (HBVLD) samples (Wilcoxon rank sum test was used to compare six-CpG risk score between two groups). **(E)** Area under the receiver operating characteristic curve (AUROC) of the six-CpG-scorer and its component CpGs. Data were AUROC (95% CI). HCC, hepatocellular carcinoma; BCLC, Barcelona Clinic Liver Cancer staging system; LASSO, least absolute shrinkage and selection operator method.

On the basis of our previous studies (9), we portrayed differentially methylated CpGs between CHB and each of the HCC phases utilizing Limma package with Bonferroni correction. The number of specifically methylated CpGs between CHB and each of the HCC phases included 2,285 for BCLC-0; 2,233 for BCLC-A; 3,345 for BCLC-B; and 23,596 for BCLC-C. There were 326 differentially methylated CpGs that could specifically distinguish early HCC (BCLC-0 and BCLC-A stages) from CHB (**Figure 2A**). Moreover, 20 robust CpG differentially methylated sites were used in this study from 33 CpGs (|delta beta |≥0.2). Meanwhile, 36 differentially methylated CpGs (|delta beta |≥0.2) could specifically distinguish early HCC from LC using paired t tests with Bonferroni correction, and 17 CpGs were selected for this study (unpublished work). In all, methylation ratios of 34 CpGs (three CpGs overlap) were investigated using MBS in the HBVLD and early HCC samples (**Supplementary Tables S2**, **S3**).

Thirty-four CpGs were reduced to six potential predictors using the LASSO regression model. The cross-validated error plot and a coefficient profile plot of the LASSO regression model were produced (**Figures 2B, C**). The logistic regression was used for building the six-CpG-scorer: where risk score = −0.87 – 3.73 × cg14171514 + 2.58 × cg07721852 + 6.91 × cg05166871 − 9.85× cg18087306 + 4.50 × cg05213896 + 4.39 × cg18772205, where values of each CpG were methylation ratio measured from MBS. We then applied this formula to calculate the risk score for early HCC of each patient based on their individual six CpG methylation ratio. The risk score was significantly increased in the early HCC samples versus the HBVLD samples (*p* < 2.22 × 10^−16^) (**Figure 2D**). The six CpGs and their combination six-CpG-scorer also showed diagnostic accuracy (**Figure 2E** and **Supplementary Table S4**). According to determining of maximum Youden index, 0 severed as the optimal cutoff point of risk score. Therefore, we classified those patients with risk score < 0 as low-risk group, and those with risk score ≥ 0 as high-risk group. The distribution of demographic and clinical characteristics did not vary significantly between the high-risk and low-risk group (**Supplementary Table S5**).

### Six-CpG-Scorer Signature Was Independent of Clinical Factors

The univariate logistic analysis was performed in 10,000 bootstrap datasets. Six of the 18 candidate variables indicated a higher early HCC risk; among these were higher age (≥43 years), male, individuals with higher AFP, higher six-CpG-scorer, lower TP (<65 g/L), and lower TBil (**Table 2**).

**Table 2:** All 18 variables included in the backwards feature selection analysis.

Variable	Univariable		Multivariable	
	OR (95% CI)	*p*-value	OR (95% CI)	*p*-value
Age (years), ≥43 *vs*. <43	5.07 (3.07–8.56)	1.75e–07***	5.23 (3.24–8.63)	2.56e-08***
Sex (Female *vs*. Male)	0.32 (0.17–0.56)	0.0011**	0.32 (0.19–0.53)	0.000248***
Alpha-fetoprotein (AFP) (ng/ml), log_10_^a^	1.50 (1.35–1.69)	1.85e–09***	1.48 (1.34–1.64)	3.05e-10***
Six-CpG-Scorer	2.37(1.83 –3.12)	1.10e–07***	2.58 (2.01–3.36)	1.08e-09***
Total protein(g/L), ≥65 *vs*. <65	0.38 (0.19–0.71)	0.01209*	0.32 (0.20–0.51)	0.56
Total bilirubin(μmol/L), ≥21 *vs*. <21	0.32 (0.14–0.69)	0.01588*	0.45 (0.32–0.64)	0.78
AST (U/L), log_10_	0.77 (0.45–1.28)	0.40551		
ALT (U/L), log_10_	0.51(0.43–1.06)	0.15320		
Direct bilirubin (μmol/L), ≥7 *vs*. <7	0.55 (0.77–2.35)	0.38832		
γ-GT (U/L), ≥45 *vs*. <45	1.15 (0.83–1.61)	0.47649		
Albumin (g/L), ≥40 *vs*. <40	0.74 (0.41–1.36)	0.42147		
Alkaline phosphatase (U/L), log_10_	0.77 (0.44–1.35)	0.44623		
Hemoglobin (g/L), ≥115 *vs*. <115	1.68 (0.91–3.14)	0.16827		
White blood cell count × 10^9^/L, log_10_	0.84 (0.17–4.24)	0.85430		
Monocyte count × 10^9^/L, truncate_99 + log_10_^b^	2.29 (1.14–4.67)	0.05410.		
Platelet count × 10^9^/L, ≥125 *vs*. <125	0.84(0.50–1.42)	0.58796		
Lymphocyte count × 10^9^/L, ≥1.1 *vs*. <1.1	0.82 (0.46–1.49)	0.58026		
Neutrophil count× 10^9^/L, log_10_	1.08 (0.37–2.86)	0.89871		

^a^Nonlinear transformation.

^b^Monocyte count variable was truncated with 1st and 99th percentiles and then performed with nonlinear transformation.

OR, odds ratio; γ-GT, γ-glutamyltranspeptidase; ALT, alanine aminotransferase; AST, aspartate aminotransferase. Signif. codes: “***” 0.001, “**” 0.01, “*” 0.05.

Four variables were selected by the backward feature selection procedure in 10,000 bootstrap datasets (**Table 2**). Age, sex, AFP, and six-CpG-scorer signature were independent risk factors for early HCC. The six-CpG-scorer also showed significantly higher predictive accuracy than AFP (**Supplementary Table S6**) and other demographic and clinical risk factors.

### Development of an Individualized Early HCC Diagnosis Nomogram

**Figure 3:**
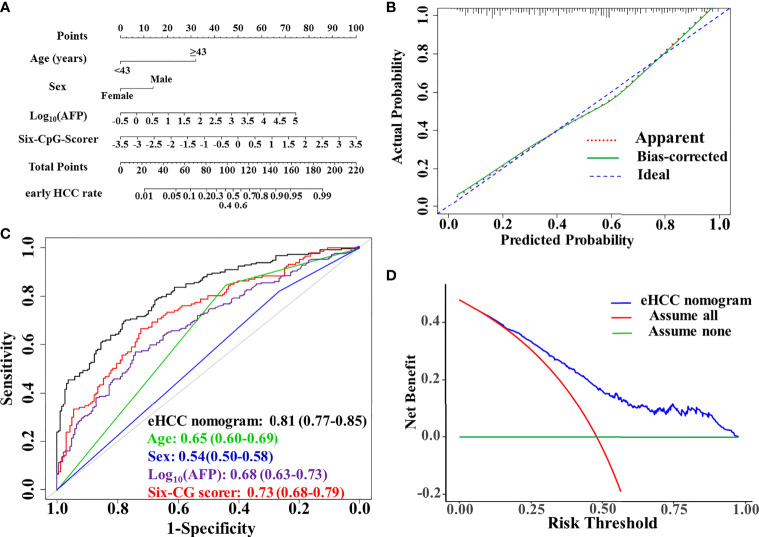
Performance evaluation and clinical usefulness of the eHCC nomogram. **(A)** Nomogram was based on age, sex, alpha-fetoprotein (AFP), and the six-CpG-scorer. **(B)** Calibration curves of the eHCC nomogram in the training dataset. The *x*-axis represented the predicted probability of early HCC risk from nomogram. The *y*-axis represented the actual early HCC rate. The blue diagonal dotted line represented a perfect performance by an ideal nomogram. The red dotted line represented the performance of the eHCC nomogram; the green solid line represented bootstrap-corrected performance of eHCC nomogram. **(C)** Area under the receiver operating characteristic curve (AUROC) of eHCC nomogram in training dataset. Data were AUROC (95% CI). **(D)** Decision curve analysis (DCA) of the nomogram predicted early HCC in the training set. The *x*-axis indicated the threshold probability and the *y*-axis indicated the net benefit. The blue curve represented the eHCC nomogram. The red line indicated the assumption that all patients were early HCC. The horizontal green line indicated the assumption that there were early HCC. eHCC, early hepatocellular carcinoma.

The early HCC diagnosis model incorporated the four risk predictors and estimated on the 20 complete datasets according to Rubin’s Rule. The prognostic index *X* (based on logistic regression model coefficients) was: *X* = −1.0944708 – 0.7183741 × Sex (Male = 0, Female = 1) + 1.7286974 × Age [(<43 years) = 0, (≥ 43 years) = 1)] + 0.2761166 × lg(AFP) + 0.7902764 × six-CpG-scorer. The calculation of the predicted risk of early HCC from HBVLD was as follows: risk of $\text{eHCC}=\frac{1}{1+\exp(-X)}$ . It presented as the early HCC nomogram (eHCC nomogram) (**Figure 3A**).

### Estimation for the C-Statistics and Brier Score by Bootstrap Validation

Internal validation was performed using the 500 times resampling enhanced bootstrap method from each of the 20 complete datasets. The result showed negligible model optimism. The apparent C-statistics and apparent Brier score was 0.805 and 0.200, respectively. The optimism of the C-statistics and Brier score was −0.0042 and 0.00164, respectively. The adjusted C-statistics and Brier score was 0.809 and 0.199, respectively.

### Diagnostic Performance and Clinical Usefulness of eHCC Nomogram

The AUROC of the model was 0.81 (95% CI, 0.77–0.85) in the training set. The sensitivity, specificity, positive predictive value (PPV), and negative predictive value (NPV) when used in differentiating the early HCC from HBVLD were 70.0%, 77.8%, 74.4%, and 73.6%, respectively. The calibration curve of the eHCC nomogram for the probability of early HCC demonstrated good agreement between prediction and observation in the training set (**Figure 3B**). The decision curve analysis of the eHCC nomogram and that for the model without the six-CpG-scorer is presented in **Figure 3C**. The DCA showed that if the threshold probability of a patient or doctor was >10%, using eHCC nomogram to predict early HCC adds more benefit than either the treat-all-patients scheme or the treat-none scheme (**Figure 3D**).

### Diagnostic Performance of the eHCC Nomogram in the Test Set

**Figure 4:**
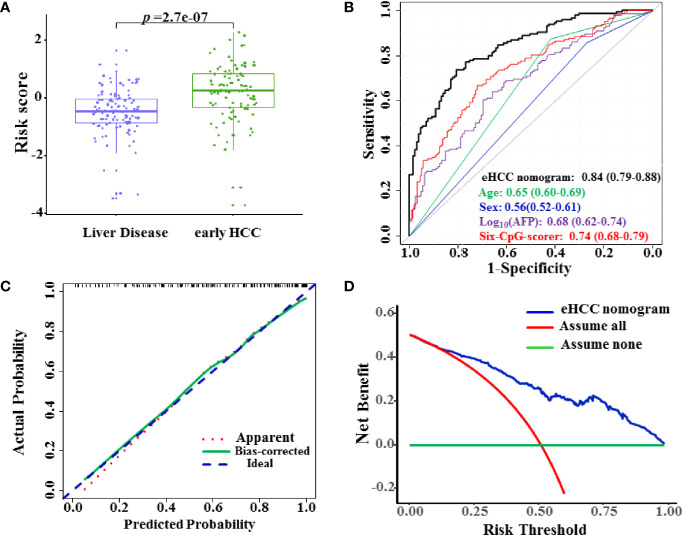
Diagnostic performance of the eHCC nomogram in the test set. **(A)** The risk score was significantly increased in the early HCC samples versus the HBVLD group (*p* = 2.7×10^–7^). **(B)** Area under the receiver operating characteristic curve (AUROC) of eHCC nomogram in the test set. Data were AUROC (95% CI). **(C)** Calibration curves of the eHCC nomogram in the test set. The *x*-axis represented the predicted probability of HCC risk from nomogram. The *y*-axis represented the actual HCC rate. The blue diagonal dotted line represented a perfect performance by an ideal nomogram. The red dotted line represented the performance of the eHCC nomogram, and the green solid line represented bootstrap-corrected performance of eHCC nomogram. **(D)** Decision curve analysis (DCA) of the nomogram predicted early HCC in the test set. The *x*-axis indicated the threshold probability, and the *y*-axis indicated the net benefit. The blue curve represented the eHCC nomogram. The red line indicated the assumption that all patients were HCC. The horizontal green line indicated the assumption that there were early HCC. eHCC, early hepatocellular carcinoma.

We further enrolled 212 patients including 111 HBVLD and 101 early HCC to serve as the test set for validation of the diagnostic potential. The risk score was significantly increased in the early HCC versus the HBVLD group in the test set (*p* = 2.7×10^–7^) (**Figure 4A**). The eHCC nomogram achieved an AUROC value of 0.84 (95% CI 0.79–0.88) between the early HCC and HBVLD (**Figure 4B**). The calibration curve demonstrated good agreement between prediction and observation in early HCC (**Figure 4C**). The sensitivity, specificity, PPV, and NPV were used in differentiating the early HCC from HBVLD and were 68.9%, 82.9%, 80.0%, and 71.8%, respectively. The nomogram also indicated good clinical benefits in DCA, which suggested an obvious diagnosis efficacy for early HCC from HBVLD (**Figure 4D**).

## Discussion

The identification of differentially methylated CpG genome-wide from the Methylation Chip (450K and EPIC) often required a test and/or replication in extra cohorts. The pyrosequencing, bisulfite-conversion-based methylation PCR, PCR cloning, and Sanger sequencing method were laborious and involved fussy work for detection methylation of each CpG site and inevitable increase of inter-batch differences in large samples. The deep sequencing of specific target CpGs often called for MBS, which is based on the NGS method to assess the methylation status at target DNA regions and with very high coverage (13). The important and commendable characteristics of MBS were low cost, low DNA input, and high repeatability and scalability. In addition, the MBS was compatible with common NGS platforms using standard equipment, making it available to most laboratories (23). Construction of amplicon libraries according to a target CpG panel was a key step of MBS. In multiplex PCR using bisulfite converted DNA as template, mixed-base primers, whose pyrimidine base Y contains a “mixture” of cytosines and thymines at the cytosine site and whose purine base R contains a mixture of guanines and adenines at the guanine site, greatly affected the amplification efficiency and led to a decrease in library quality. In the design of this study, we applied 34 “CpG-free” primer pairs (i.e., primers that do not bind to a region containing CpG dinucleotides) targeting each region covering candidate CpGs, which had been considered to produce “non-preferential” amplification of bisulfite-converted DNA genome (24). We successfully establish a version of the “CpG-free” primer pair panel applied in MBS to simultaneously investigate the methylation status of 34 candidate CpGs across a large sample size (*n* = 654). The complete and accurate methylation data of 34 candidate CpGs allowed us to screen out and integrate into a six-CpG-scorer with application of LASSO regression.

CpG methylation served as an apparatus focused on non-invasive biomarkers for chronic diseases {age-related diabetes (25), diabetic embryopathy (26), coronary heart disease (27), nonalcoholic fatty liver disease (28), or cancer [bladder cancer (29), rectal cancer (30), and HCC (31)]} and had been set up by compelling studies. Our recent study looked at CpG methylation alterations of HBV-related liver disease and developed a model discriminating compensated cirrhosis patients from CHB and decompensated cirrhosis ones based on two CpG biomarkers (32). Our previous study also supported the feasibility of using 350 CpGs for detection of each stages of HCC from healthy control (9). In this study, we successfully established and validated a novel diagnostic nomogram based on the six-CpG-scorer to distinguish early HCC patients from CHB and LC ones. Furthermore, this proposed six-CpG-scorer can diagnose the early HCC better than other demographic and characteristics risk factors. Meanwhile, eHCC nomogram validated considerable diagnostic potential to early HCC and indicated good clinical benefits in DCA.

The CpGs serving as independent risk factors of HCC from mining analysis of Methylation Chip data might point out a novel trace for the exploration of HCC progress mechanism. Based on the Illumina Human Beadchip 27K array, sphingomyelin phosphodiesterase 3 (SMPD3) and neurofilament, heavy polypeptide (NEFH) were found to behave as tumor suppressor genes in HCC after validation *in vitro* and *in vivo* (33). Gentilini et al. applied the Methylation 450K BeadChip array and showed that four epigenetically regulated candidate genes [adherens junctions associated protein 1 (AJAP1), adenosine deaminase RNA specific B2 (ADARB2), protein tyrosine phosphatase receptor type N2 (PTPRN2), and sidekick cell adhesion molecule 1 (SDK1)] were potentially involved in the pathogenesis of HCC (34). The finding of AJAP1 was consistent with clinical observation of low AJAP1 expression as an independent factor for predicting disease-free survival (DFS) (35). Mechanically, AJAP1 could block epithelial-to-mesenchymal transition (EMT) *via* suppressing β-catenin/zinc finger E-box binding homeobox 1 (ZEB1) signaling.

In this study, cg14171514 was located within the 5’UTR of the neuroblast differentiation-associated protein (AHNAK) gene, and significantly hypomethylated both in the PBMC DNA of early HCC patients compared with HBVLD ones in this study. Consistently, the aberrant hypomethylation status in the AHNAK gene promoter region from HCC patients’ PBMC DNA was verified in methylation-specific PCR (MSP) results of our previous studies (36). Aberrant AHNAK methylation level in PBMC DNA was associated with HCC. In addition, AHNAK mRNA had been reported to be overexpressed in liver cancer tissues by qPCR (36) and the cancer genome atlas (TCGA) data (**Supplementary Figure S1**). Our novel results from immunoprecipitation in combination with mass spectrometry (IP-MS) analysis strongly indicated that AHNAK protein was involved in and promoted HCC progress (data not published). The cg18087306 was located within the body of Lamin B2 (LMNB2) gene, whose expression level in HCC tissue was higher relative to peritumor tissue. LMNB2 was a promising HCC prognostic and diagnostic biomarker (37).

## Conclusions

We had shown characteristic changes of 34 CpGs in HBVLD and early HCC across a clinical cohort, identified the six-CpG-scorer, and validated their diagnostic efficacy. Thus, we proposed that the six-CpG-scorer-based nomogram is a potential non-invasive diagnostic tool for early HCC from HBVLD, and performed internal validation using the 500 times resampling enhanced bootstrap method from each of the 20 complete datasets to evaluate the stability and then applied it to the test set for validation. The external validation of the monogram will be needed in much larger cohorts from different centers or regions to promote the efficacy and stability to further benefit HCC populations.

## Data Availability Statement

The datasets presented in this study can be found in online repositories. The names of the repository/repositories and accession number(s) can be found in the article/**Supplementary Material**.

## Ethics Statement

The studies involving human participants were reviewed and approved by Ethics Committee of the Beijing You’An Hospital. The patients/participants provided their written informed consent to participate in this study.

## Author Contributions

YHZ and KL conceived and designed the experiments. KL, YS, LQ, and AL performed all of the experiments. YS designed multiplex bisulfite sequencing and acquired raw data. KL, LR, and SJ conducted the statistical analysis. KL, LQ, CZ, JS, and YZ contributed to collection and processing of samples. All authors contributed to the article and approved the submitted version.

## Funding

This project was supported by grants from the National Key R&D Program of China (2020YFE0202400), Beijing Natural Science Foundation (7202069 and 7191004), Capital’s Funds of Health Improvement and Research (CFH2020-1-2182 and CFH2020-2-1153), Beijing Municipal Science & Technology Commission (Z171100001017078), and Beijing Key Laboratory (BZ0373).

## Conflict of Interest

The authors declare that the research was conducted in the absence of any commercial or financial relationships that could be construed as a potential conflict of interest.

## Publisher’s Note

All claims expressed in this article are solely those of the authors and do not necessarily represent those of their affiliated organizations, or those of the publisher, the editors and the reviewers. Any product that may be evaluated in this article, or claim that may be made by its manufacturer, is not guaranteed or endorsed by the publisher.
