# Supplementary material for: A CpG Methylation Signature as a Potential Marker for Early Diagnosis of Hepatocellular Carcinoma From HBV-Related Liver Disease Using Multiplex Bisulfite Sequencing
Source: Front Oncol. 2021 Oct 20;11:756326. doi: 10.3389/fonc.2021.756326 (PMC8564137; doi:10.3389/fonc.2021.756326)
Supplement: Supplementary file 1 [file Presentation_1.pptx]

## Slide 1
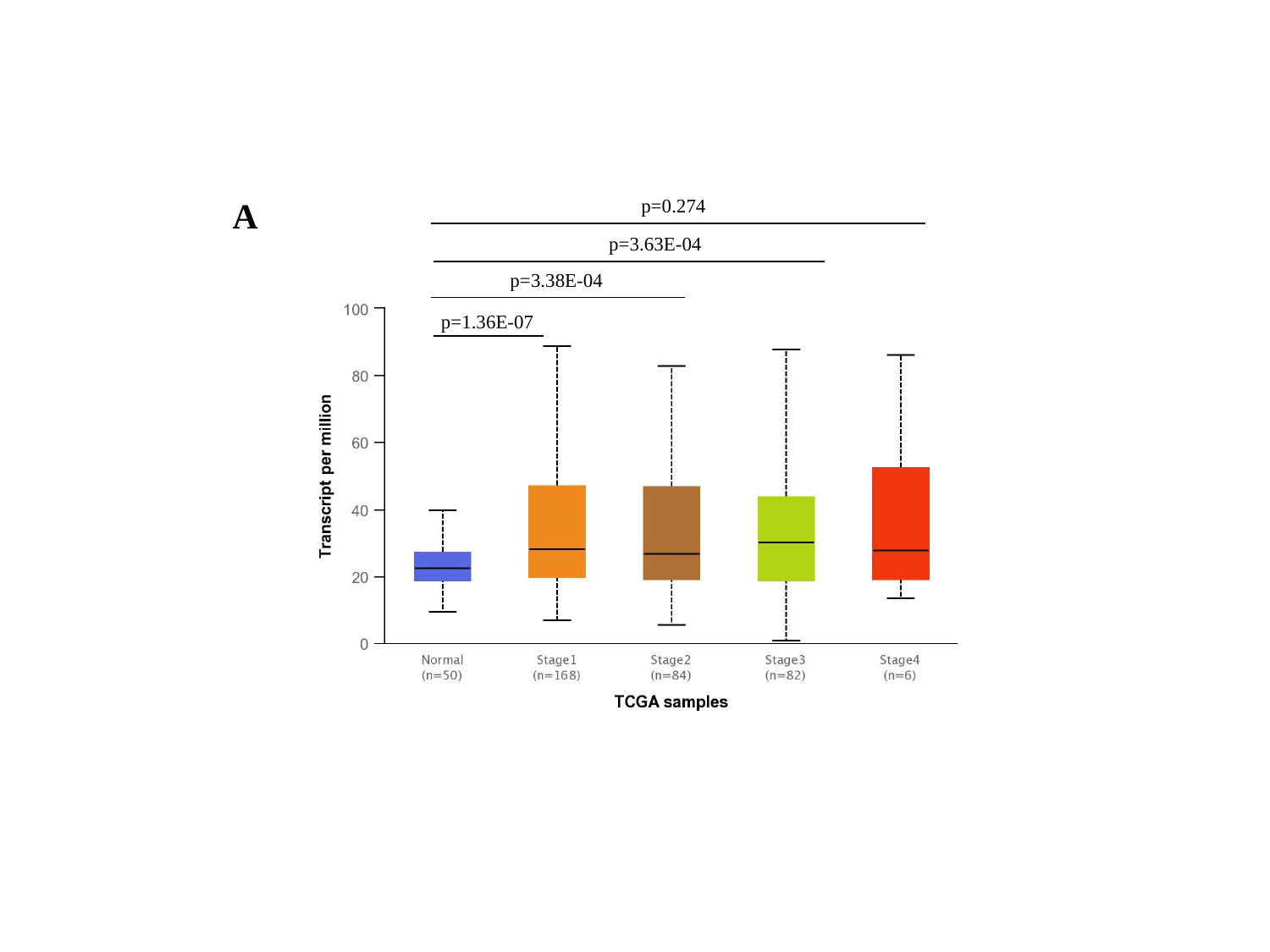

A
p=0.274
p=3.63E-04
p=3.38E-04
p=1.36E-07

## Slide 2
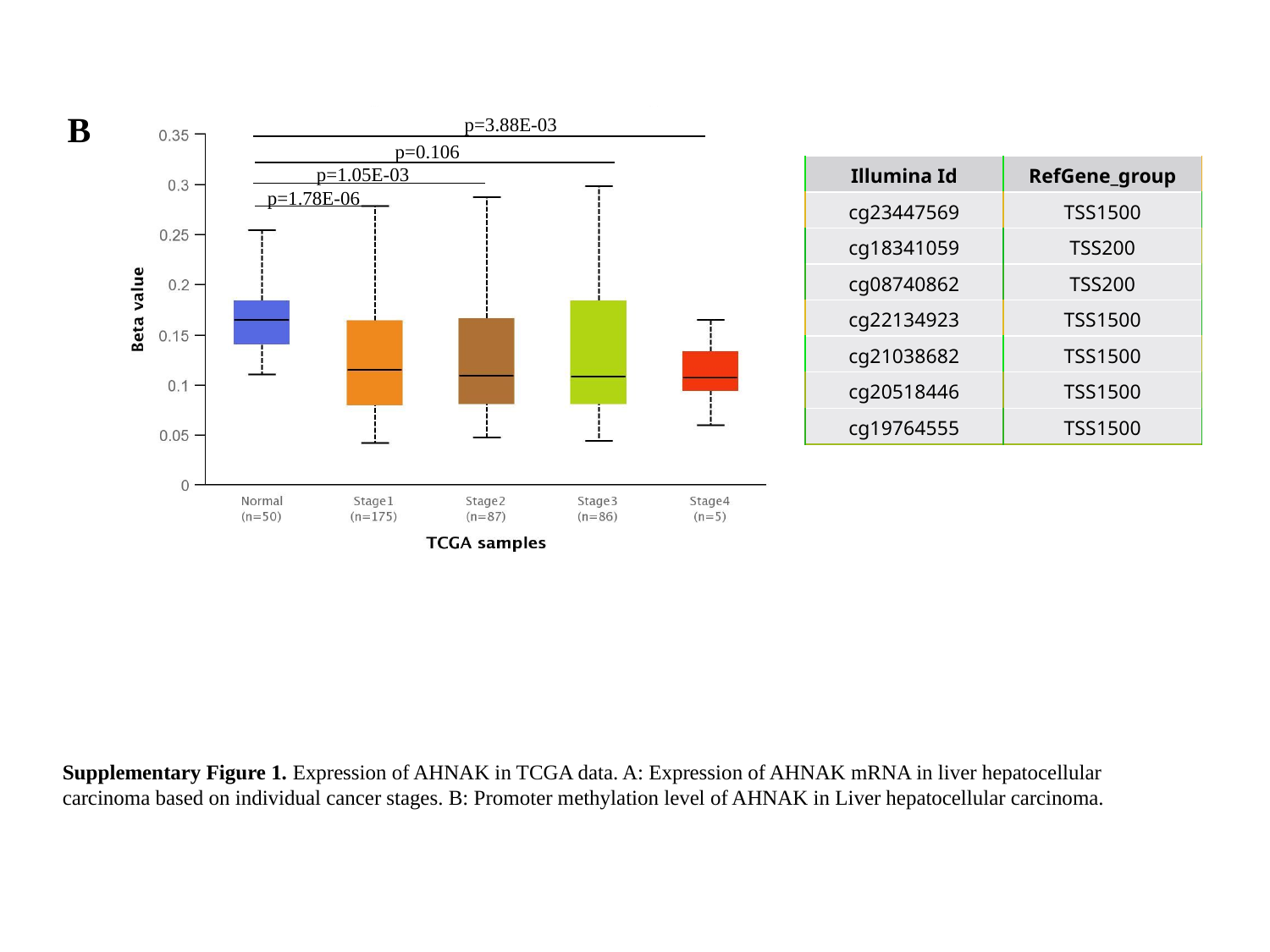

B
p=3.88E-03
p=0.106
p=1.05E-03
| Illumina Id | RefGene\_group |
| --- | --- |
| cg23447569 | TSS1500 |
| cg18341059 | TSS200 |
| cg08740862 | TSS200 |
| cg22134923 | TSS1500 |
| cg21038682 | TSS1500 |
| cg20518446 | TSS1500 |
| cg19764555 | TSS1500 |
p=1.78E-06
Supplementary Figure 1. Expression of AHNAK in TCGA data. A: Expression of AHNAK mRNA in liver hepatocellular carcinoma based on individual cancer stages. B: Promoter methylation level of AHNAK in Liver hepatocellular carcinoma.
