# Supplementary material for: A CpG Methylation Signature as a Potential Marker for Early Diagnosis of Hepatocellular Carcinoma From HBV-Related Liver Disease Using Multiplex Bisulfite Sequencing
Source: Front Oncol. 2021 Oct 20;11:756326. doi: 10.3389/fonc.2021.756326 (PMC8564137; doi:10.3389/fonc.2021.756326)
Supplement: Supplementary file 2 [file Table_1.doc]

Supplementary Table 1. The nonlinear effects test between 17 continuous independent variables and early HCC.

| Variables | Chi-Squre | df | *P* value for nonlinear |
| --- | --- | --- | --- |
| Age | 46.11 | 2 | 0.0174 |
| Log(ALT) | 8.74 | 2 | 0.629 |
| Log(AST) | 6.89 | 2 | 0.105 |
| TBil | 6.68 | 2 | 0.692 |
| DBil | 6.75 | 2 | 0.034 |
| TP | 5.54 | 2 | 0.039 |
| ALB | 12.15 | 2 | 0.008 |
| γ-GT | 2.68 | 2 | 0.121 |
| Log(ALP) | 0.73 | 2 | 0.393 |
| Log(WBC) | 0.59 | 2 | 0.626 |
| hemoglobin | 6.31 | 2 | 0.025 |
| PLT | 0.44 | 2 | 0.909 |
| LYM | 6.73 | 2 | 0.902 |
| Log(NEUT) | 3.67 | 2 | 0.289 |
| Log(MONO) | 1.03 | 2 | 0.589 |
| Log(AFP) | 36. 06 | 2 | 0.850 |
| Six-CpG-Scorer | 53.08 | 2 | 0.479 |
