# Supplementary material for: A CpG Methylation Signature as a Potential Marker for Early Diagnosis of Hepatocellular Carcinoma From HBV-Related Liver Disease Using Multiplex Bisulfite Sequencing
Source: Front Oncol. 2021 Oct 20;11:756326. doi: 10.3389/fonc.2021.756326 (PMC8564137; doi:10.3389/fonc.2021.756326)
Supplement: Supplementary file 4 [file Table_3.docx]

| Supplementary Table 3. Methylation ration of 34 CpG sites in HBVLD and early HCC group in training set | | | |
| --- | --- | --- | --- |
| CpGs | HBVLD ( mean±SD) | early HCC (mean±SD) | *p* |
| cg04998202 | 0.38±0.14 | 0.40±0.13 | 0.16 |
| cg20253872 | 0.39±0.31 | 0.42±0.23 | 0.11 |
| cg05702218 | 0.37±0.17 | 0.38±0.26 | 0.96 |
| cg12467404 | 0.65±0.15 | 0.68±0.12 | 0.012 |
| cg14171514 | 0.28±0.17 | 0.22±0.11 | 6.4×10^-5^ |
| cg07721852 | 0.11±0.10 | 0.12±0.10 | 2.2×10^-4^ |
| cg03734874 | 0.09±0.07 | 0.10±0.06 | 0.19 |
| cg05891094 | 0.94±0.15 | 0.92±0.14 | 0.51 |
| cg21402921 | 0.02±0.02 | 0.03±0.03 | 7.1×10^-4^ |
| cg04484415 | 0.30±0.35 | 0.33±0.27 | 0.32 |
| cg27395066 | 0.21±0.18 | 0.24±0.15 | 0.13 |
| cg05166871 | 0.06±0.05 | 0.10±0.10 | 2.2×10^-4^ |
| cg11783901 | 0.66±0.15 | 0.64±0.15 | 0.036 |
| cg22632947 | 0.44±0.34 | 0.53±0.25 | 4.7×10^-16^ |
| cg02185248 | 0.18±0.20 | 0.28±0.18 | 0.9 |
| cg18087306 | 0.08±0.03 | 0.08±0.05 | 6.4×10^-7^ |
| cg05213896 | 0.12±0.05 | 0.14±0.06 | 6.1×10^-3^ |
| cg09404516 | 0.33±0.28 | 0.42±0.24 | 0.0046 |
| cg09778596 | 0.40±0.11 | 0.41±0.17 | 0.73 |
| cg01620164 | 0.44±0.14 | 0.44±0.13 | 0.75 |
| cg15462501 | 0.15±0.12 | 0.18±0.11 | 0.014 |
| cg25635352 | 0.03±0.03 | 0.04±0.03 | 0.083 |
| cg18772205 | 0.23±0.07 | 0.26±0.07 | 2.3×10^-5^ |
| cg09470983 | 0.61±0.11 | 0.59±0.11 | 0.016 |
| cg04398282 | 0.49±0.12 | 0.49±0.12 | 0.81 |
| cg17588578 | 0.45±0.36 | 0.50±0.23 | 0.47 |
| cg15747825 | 0.27±0.10 | 0.27±0.12 | 0.70 |
| cg14279856 | 0.64±0.13 | 0.63±0.14 | 0.52 |
| cg20445774 | 0.22±0.16 | 0.23±0.17 | 0.52 |
| cg04749631 | 0.47±0.26 | 0.23±0.14 | 0.14 |
| cg04115680 | 0.27±0.10 | 0.28±0.08 | 0.16 |
| cg21183256 | 0.17±0.11 | 0.20±0.16 | 0.054 |
| cg27616227 | 0.25±0.13 | 0.28±0.12 | 0.019 |
| cg02243522 | 0.36±0.15 | 0.40±0.14 | 0.011 |
| six-CpG-scorer | -0.31±0.36 | 0.04±0.10 | 2.2×10^-16^ |

Abbreviations: HBVLD, HBV-related liver disease
