# Supplementary material for: A CpG Methylation Signature as a Potential Marker for Early Diagnosis of Hepatocellular Carcinoma From HBV-Related Liver Disease Using Multiplex Bisulfite Sequencing
Source: Front Oncol. 2021 Oct 20;11:756326. doi: 10.3389/fonc.2021.756326 (PMC8564137; doi:10.3389/fonc.2021.756326)
Supplement: Supplementary file 5 [file Table_4.doc]

| Supplementary Table 4 Univariate association of 34 CpGs with early HCC | | | |
| --- | --- | --- | --- |
| CpGs | *p* | **OR (95% CI)** | AUROC (95% CI) |
| cg01620164 | 0.64 | 0.66(0.15-2.77) | 0.53 (0.47-0.59) |
| cg02243522 | 0.0362 * | 5.83(1.48-23.79) | 0.57(0.50-0.634) |
| cg03734874 | 0.10 | 14.67(1.02-242.77) | 0.59 (0.53-0.66) |
| cg04115680 | 0.45 | 2.38(0.36-15.96) | 0.55(0.48-0.62) |
| cg04398282 | 0.54 | 1.75(0.39-7.83) | 0.54(0.47-0.60) |
| cg04998202 | 0.31 | 2.27(0.60-8.74) | 0.55(0.48-0.61) |
| **cg05166871** | **1.92e-06 ***** | **5.77(3.21-12.54)** | **0.70(6.42-0.76)** |
| **cg05213896** | **0.56** | **3.02(0.13-7.22)** | **0.52(0.46-0.59)** |
| **cg07721852** | **0.0105 *** | **2.30(0.33-18.77)** | **0.65(0.59-0.71)** |
| cg09470983 | 0.0313 * | 0.11(0.02-0.58) | 0.57(0.51-0.64) |
| cg09778596 | 0.584 | 1.57(0.40-6.23) | 0.533(0.47-0.59) |
| cg11783901 | 0.04 * | 0.18(0.04-0.69) | 0.56(0.49-0.63) |
| cg12467404 | 0.166 | 3.40(0.81-14.94) | 0.56(0.49-0.62) |
| **cg14171514** | **0.000675 ***** | **0.06(0.01-0.22)** | **0.60(0.54-0.67)** |
| cg14279856 | 0.19 | 0.31(0.06-1.33) | 0.56(0.49-0.62) |
| cg15747825 | 0.79 | 0.77(0.15-3.97) | 0.52 (0.45-0.59) |
| **cg18087306** | **0.54** | **0.19 (0.00-1.57)** | **0.53 (0.47-0.59)** |
| **cg18772205** | **0.00391 **** | **8.01 (6.92-10.48)** | **0.61 (0.55-0.68)** |
| cg20445774 | 0.58 | 1.45 (0.81-1.44) | 0.52(0.45-0.58) |
| cg21183256 | 0.22 | 2.50 (0.74-8.8) | 0.58 (0.50-0.63) |
| cg21402921 | 0.0394 * | 5.38 (1.63-6.88) | 0.63 (0.57-0.69) |
| cg25635352 | 0.0688 . | 4.20 (1.93-11.21) | 0.63 (0.57-0.70) |
| cg27395066 | 0.0817 . | 3.40 (1.09-11.11) | 0.59 (0.53-0.66) |
| cg27616227 | 0.01415 * | 8.19 (2.81-35.39) | 0.59 (0.53-0.66) |
| cg04484415 | 0.89 | 0.95 (0.49-1.81) | 0.53 (0.46-0.60) |
| cg04749631 | 0.21 | 1.83 (0.84-4.04) | 0.56 (0.49-0.63) |
| cg05702218 | 0.61 | 0.83 (0.45-1.52) | 0.52(0.45-0.59) |
| cg05891094 | 0.91 | 0.19 (0.03-0.91) | 0.66 (0.59-0.72) |
| cg15462501 | 0.01181 * | 1.28 (0.25-6.90) | 0.62 (0.56-0.68) |
| cg17588578 | 0.47 | 1.34 (0.69-2.62) | 0.47 (0.39-0.54) |
| cg20253872 | 0.34 | 1.49 (0.74-3.05) | 0.55(0.48-0.62) |
| cg22632947 | 0.0423 * | 2.28 (1.18-4.83) | 0.59 (0.52-0.66) |
| cg02185248 | 0.000523 *** | 8.18 (3.09-22.81) | 0.67 (0.61-0.73) |
| cg09404516 | 0.00882 ** | 3.26 (1.57-6.94) | 0.61 (0.55-0.68) |
| **Six-CpG-scorer** | **5.11e-11** | **2.97(2.28-3.95)** | **0.73 (0.68-0.79)** |

The bold represented the selected six CpG sites and their combination six-CpG-scorer. Abbreviations: OR, odds ratio; AUROC, area under the receiver operating characteristic curve. Signif. codes: ‘***’ 0.001, ‘**’ 0.01, ‘*’ 0.05.
