# Supplementary material for: A CpG Methylation Signature as a Potential Marker for Early Diagnosis of Hepatocellular Carcinoma From HBV-Related Liver Disease Using Multiplex Bisulfite Sequencing
Source: Front Oncol. 2021 Oct 20;11:756326. doi: 10.3389/fonc.2021.756326 (PMC8564137; doi:10.3389/fonc.2021.756326)
Supplement: Supplementary file 6 [file Table_5.doc]

| Supplementary Table 5: Baseline characteristics of patients by six-CpG-scorer assessment set | | | | | | | |
| --- | --- | --- | --- | --- | --- | --- | --- |
|  |  | Training set (n=442) | |  |  | Test set (n=212) | |
|  | Number of  patients | Low risk (< 0) | High risk (≥ 0) |  | Number of  patients | Low risk (< 0 ) | High risk (≥ 0) |
| Age(year)  <43  ≥43 | 127  315 | 85(66.9%)  165(52.4%) | 42(33.1%)  150(47.6%) |  | 60  152 | 43(71.7%)  80(52.6%) | 17(28.3%)  72(47.4%) |
| Sex  Male  Female | 344  98 | 187(54.4%)  63(64.3%) | 157(45.6%)  35(35.7%) |  | 170  42 | 91(53.5%)  32(76.2%) | 79(46.5%)  10(23.8%) |
| ALT (U/L)  <40  ≥40 | 236  204 | 131(55.5%)  118(57.8%) | 105(44.5%)  86(42.2%) |  | 111  101 | 60(54.1%)  63(62.4%) | 51(45.9%)  38(37.6%) |
| AST (U/L)  <40  ≥40 | 265  175 | 153(57.7%)  96(54.9%) | 112(42.3%)  79(45.1%) |  | 114  98 | 67(58.8%)  56(57.1%) | 47(41.2%)  42(42.9%) |
| Total bilirubin (μmol/L)  <21  ≥21 | 290  150 | 162(55.9%)  87(58.0%) | 128(44.1%)  63(42.0%) |  | 142  70 | 82(57.7%)  41(58.6%) | 60(42.3%)  29(41.4%) |
| Direct bilirubin (μmol/L)  <7  ≥7 | 345  95 | 194(56.2%)  55(57.9%) | 151(43.8%)  40(42.1%) |  | 160  52 | 92(57.5%)  31(59.6%) | 68(42.5%)  21(40.4%) |
| Total protein (g/L)  <65  ≥65 | 303  137 | 169(55.8%)  79(57.7%) | 134(44.2%)  58(42.3%) |  | 150  62 | 88(58.7%)  35(56.5%) | 62(41.3%)  27(43.5%) |
| Albumin (g/L)  <40  ≥40 | 243  197 | 137(56.4%)  113(57.4%) | 106(43.6%)  84(42.6%) |  | 117  95 | 64(54.7%)  59(62.1%) | 53(45.3%)  36(37.9%) |
| γ-GT (U/L)  < 45  ≥45 | 210  197 | 127(60.5%)  103(52.3%) | 83(39.5%)  94(47,7%) |  | 72  133 | 47(65.3%)  72(54.1%) | 25(34.7%)  61(45.9%) |
| Alkaline phosphatase (U/L)  ≤ 100  >100 | 301  107 | 172(57.1%)  58(54.2%) | 129(42.9%)  49(45.8%) |  | 90  100 | 62(68.9%)  69(69.0%) | 28(31.1%)  31(31.0%) |
| WBC count × 10^9^/L  < 3.5  3.5-9.5  >9.5 | 330  89  22 | 181(54.8%)  56(62.9%)  12(54.5%) | 149(45.2%)  33(37.1%)  10(45.5%) |  | 153  38  21 | 88(57.5%)  25(65.8%)  10(47.6%) | 65(42.5%)  13(34.2%)  11(52.4%) |
| Hemoglobin  (g/L)  < 130  ≥130 | 281  160 | 152(54.1%)  97(60.6%) | 129(45.9%)  63(39.4%) |  | 137  75 | 76(55.5%)  47(62.7%) | 61(44.5%)  28(37.3%) |
| Platelet count × 10^9^/L  <125  ≥ 125 | 236  206 | 128(54,2%)  122(59.2%) | 108(45.8%)  84(40.8%) |  | 128  84 | 72(56.3%)  51(60.7%) | 56(43.7%)  33(39.3%) |
| Lymphocyte count×10^9^/L  <1.1  ≥1.1 | 320  121 | 182(56.9%)  68(56.2%) | 138(43.1%)  53(43.8%) |  | 143  59 | 86(60.1%)  27(45.8%) | 57(39.9%)  32(54.2%) |
| Monocyte count× 10^9^/L  < 0.6  ≥0.6 | 396  45 | 228(57.6%)  21(46.7%) | 168(42.4%)  24(53.3%) |  | 188  24 | 113(60.1%)  10(41.7%) | 75(39.9%)  14(58.3%) |
| Neutrophil count× 10^9^/L  < 1.8  1.8-6.3  >6.3 | 312  91  28 | 164(52.6%)  61(67.0%)  15(53.6%) | 148(47.4%)  30(33.0%)  13(46.4%) |  | 151  38  23 | 87(57.6%)  27(71.1%)  9(39.1%) | 64(42.4%)  11(28.9%)  14(60.9%) |
| Alpha-fetoprotein (ng/mL)  <20  ≥20 | 252  182 | 150(59.5%)  87(47.8%) | 102(40.5%)  95(52.2%) |  | 112  98 | 69(61.6%)  54(55.1%) | 43(38.4%)  44(44.9%) |

Abbreviations: HBVLD, HBV-related liver disease; CHB, chronic hepatitis B; LC, HBV-related liver cirrhosis; HCC, Hepatocellular carcinoma; BCLC, Barcelona clinic liver cancer staging system; γ-GT, γ-glutamyltranspeptidase; WBC, white blood cell. ALT, Alanine aminotransferase; AST, Aspartate aminotransferase.
