# Supplementary material for: A CpG Methylation Signature as a Potential Marker for Early Diagnosis of Hepatocellular Carcinoma From HBV-Related Liver Disease Using Multiplex Bisulfite Sequencing
Source: Front Oncol. 2021 Oct 20;11:756326. doi: 10.3389/fonc.2021.756326 (PMC8564137; doi:10.3389/fonc.2021.756326)
Supplement: Supplementary file 7 [file Table_6.docx]

| Supplementary Table 6. Comparison of accuracy and discriminative ability between six-CpG-scorer and AFP | | |
| --- | --- | --- |
|  | six-CpG-scorer | Log_10_(AFP) |
| AUROC | 0.73 (95% CI=0.68-0.79) | 0.68 (95% CI=0. 63-0.73) |
| Accuracy | 72.6% | 65.2% |
| Precision | 69.8% | 64.4% |
| Sensitivity | 68.9% | 55.6% |
| Specificity | 71.0% | 62.6% |
| Positive predictive value | 68.9% | 65.2% |
| Negative predictive value | 70.3% | 63.4% |
| AIC | 550.9 | 768.5 |

AUROC: area under the receiver operating characteristic curve; AIC: akaike information criterion
